# Body Mass Index and risk for onset of mood and anxiety disorders in the general population: Results from the Netherlands Mental Health Survey and Incidence Study-2 (NEMESIS-2)

**DOI:** 10.1186/s12888-022-04077-w

**Published:** 2022-08-02

**Authors:** Leonore de Wit, Margreet ten Have, Pim Cuijpers, Ron de Graaf

**Affiliations:** 1 Department of Clinical, Neuro and Developmental Psychology, Vrije Universiteit Amsterdam, Van der Boechorststraat 7-9, 1081 BT Amsterdam, The Netherlands; 2 Netherlands Institute of Mental Health and Addiction, Utrecht, The Netherlands

**Keywords:** Obesity, BMI, Mood, Anxiety

## Abstract

**Background:**

Examine the onset of a clinical diagnosis of mood (major depression, dysthymia and bipolar disorder)- and anxiety disorders (panic disorder, agoraphobia without panic disorder, social phobia, specific phobia and generalized anxiety disorder) by Body Mass Index levels at baseline in the general adult population over three years.

**Methods:**

Data are from NEMESIS-2, a representative psychiatric cohort study in the Netherlands. A total of 5303 subjects aged 18–64 were interviewed with the CIDI (3.0 based on DSM-IV) in two waves, with an interval of three years. The first wave was performed from November 2007 to July 2009, the second wave from November 2010 to June 2012.

**Results:**

Persons with obesity at baseline had a significantly increased risk of the onset of any mood -or anxiety disorder adjusting for covariates compared to persons with a normal Body Mass Index (OR = 1.71; 95% CI: 1.11–2.62). The odds ratio of the underweight category was non-significant. A dose–response effect of the continuous BMI scores on the onset of any mood or anxiety disorder was found (OR = 1.06; 95% CI: 1.02 = 1.10; *p* < 0.01).

**Conclusions:**

Obesity at baseline is a risk for the onset of mood -and anxiety disorders at three year follow up.

## Background

In the past decades, obesity has become a serious threat to public health worldwide [1–3]. Obesity, commonly defined [4] by a Body Mass Index (BMI) above 30 (height/kg^2^), is caused by a combination of a high level intake of energy-dense food and a low level of physical activity. This leads to an energy imbalance between calorie intake and calories burned, which in turn leads to weight gain [5]. According to the World Health Organization (WHO), the global prevalence of obesity has nearly tripled since 1975. Nowadays 13% of the worldwide adult population meet the criteria of obesity. The prevalence rates of obesity in western societies are even more alarming and vary from 10–23% in Europe to 22–25% in the United States [6, 7] Next to that, 39% of the worldwide population is overweight (BMI 25–29.9) [8].

Previous research has indicated that having an unhealthy BMI (e.g. obesity and overweight) has a negative influence on quality of life [9]. Moreover, itis associated with various chronic physical diseases such as cardiovascular disease, various types of cancer, type 2 diabetes mellitus and sleep apnea [3, 10, 11]^.^ Next to that, there is strong evidence for an association between unfavorable BMI and mental disorders, especially in depression and anxiety [12–16]. A recent meta-analysis gave convincing evidence for a U-shaped association, meaning that underweight is also associated with depression [17].

There are various explanations for the mechanisms underlying the relation between BMI and mental disorders. Both psychological as well as biological factors seem to have an influence on the association [15, 16, 18, 19]. For instance, chronic stress in persons with depression and anxiety leads dysregulation of the hypothalamic–pituitary–adrenal-axis (HPA –axis) is leading to elevated levels of cortisol which is in turn associated with both obesity and depression [16, 19, 20]. Also, inflammatory markers can be detected in persons with obesity and persons with depression and might play a role in the association [16, 19, 21, 22].

Common genes might also play a role in the association between obesity and mental disorders. For instance, a recent meta-analysis gave evidence for an interaction effect between the FTO gene, BMI and depression [23]. Another explanation is that people with obesity have low self-esteem because of their body image which might lead to depression and anxiety. Lifestyle factors such as smoking, low physical activity and sedentary behavior are also more prevalent among people with unhealthy BMI status and in people with mental disorders [24, 25].

Although it has been established in various meta-analyses that there is a cross-sectional association between obesity and mental disorders, the association between obesity and the of *onset* mental disorders in the adult general population is not clear yet [13, 14, 17]. Understanding the relationship between unhealthy BMI and mental disorders is relevant, because both conditions are highly prevalent and associated with an increased risk of morbidity and mortality [26–28]. Hence, if obese and overweight persons have an increased risk for onset of mental disorders, interventions preventing these mental disorders among them might lead to lower burden of these mental diseases for the individual and to less economic costs for societies [6, 29].

Up to now, only few longitudinal population-based studies have used standardized psychiatric assessment interviews examining the relationship between BMI status and mental disorders. Therefore, the onset of DSM clinical diagnosis of mental disorders in persons with unhealthy BMI is understudied in the general population [13, 14, 17, 30, 31]. Furthermore, most prospective studies in this field focused on the relationship between BMI levels and the onset of depression (i.e. Major depressive disorder, dysthymia, bipolar disorder) whereas the risk between obesity and the onset of anxiety disorders (i.e. panic disorder, agoraphobia without panic disorder, social phobia, specific phobia and generalized anxiety disorder), is far less investigated [13]. A recent meta-analysis of high-quality studies, found an association between both underweight and obesity and depression. However, an association of overweight and the onset of depression was only found in women, not in men [17]. Moreover, findings on the relationship between obesity and onset of anxiety disorders in the general population are mixed, with some studies showing a positive effect in women [32] or only in men [33], others showing no effect at all [6, 31].

Furthermore, longitudinal studies investigating the onset of mental disorders in persons with unfavorable BMI, have not always excluded psychopathology at baseline [14, 17, 34]. Next to that, numerous other covariates influencing the association, such as gender, age, low social economic status (SES), physical activity, somatic diseases and psychotropic medication use have been described [29, 35–37]. Most previous studies, however, have not controlled for all these covariates. Therefore, it is unclear whether the incidence of psychopathology was due to obesity [14, 17]. Next to that, most studies in the adult general population are cross-sectional and are less able to take the temporal sequence in which BMI levels and onset of mental disorders occur into account [12–14, 17].Based on the quality of these previous studies, several authors have argued that high quality longitudinal studies are needed to establish a clear view on the risk of BMI levels on the onset of a variety of common mental disorders in the adult general population [13, 14, 17, 34].

The aim of the present study is to investigate the incidence of mood and anxiety disorders by BMI levels at baseline in the adult general population. Based on research findings so far, we expect that persons with unfavorable BMI (i.e. underweight, overweight and obesity) at baseline are at risk for the onset of mood and anxiety disorders at follow up. In our design we established BMI categories at baseline in order to investigate the onset of mood and anxiety disorders three years later using a standardized psychiatric instrument, while controlling for various confounding variables. We did this in 3 different cohorts: a cohort at risk of the onset of any mood and/or anxiety disorder (12-month anxiety and mood disorders were excluded at baseline), a cohort at risk of the onset of any mood disorder only (12-month mood disorder was excluded at baseline) and a cohort at risk of the onset of any anxiety disorder only (12-month anxiety disorder was excluded at baseline). At risk means that the participants in the sample are having the possibility of the onset of mood or anxiety disorder. With this design and the fact that the study was carried out in a large, population-based sample (NEMESIS-2) we were able to overcome many of the limitations of previous studies, including the representativeness of the sample, strict diagnosis of mental disorders and the fact that we controlled for a broad range of relevant covariates (i.e. sociodemographic factors, lifestyle factors, physical disorders, psychotropic medication use and psychopathology).

## Methods

### Sample description and study design

NEMESIS-2 is a psychiatric epidemiological cohort study of the Dutch general population aged 18 to 64 years consisting of four waves in total. A more comprehensive description of the design, the sampling procedures and the instruments of the baseline and follow up wave has been provided elsewhere [38, 39].

In short, it is based on a multistage, stratified random sampling of households, with one respondent randomly selected in each household. Based on the most recent birthday at first contact within the household, an individual aged 18–64 years with sufficient fluency in the Dutch language was randomly selected. Addresses of institutions and thus institutionalized individuals (i.e. those living in hospices, prisons) were excluded. Those temporarily living in institutions, however, could be interviewed later during the fieldwork if they returned home.

The study was approved by a medical ethics committee (the Medical Ethics Review Committee for Institutions on Mental Health Care, METIGG). After having been informed about the study aims, respondents provided written informed consent at the start of each wave of interviews. The current study uses data of the first two waves. Between the first and second wave, there was an interval of three years The mean period between the interviews was 3 years and 7 days (1.102 days; *sd* = 64).

In the first wave (T0) performed from November 2007 to July 2009, a total of 6,646 persons were interviewed (response rate 65.1%; average interview duration: 95 min). This sample was nationally representative, although younger subjects were somewhat underrepresented. The interviews, collecting data on outcome variables as well as covariates in this study, were laptop computer-assisted and almost all were held at the respondent’s home. All T0 respondents were approached for follow-up, three years after T0 from November 2010 to June 2012. Of this group, 5,303 persons were interviewed again (response rate 80.4%, with those deceased excluded; average interview duration: 84 min). Attrition rate was not significantly associated with all main categories and individual 12-month mental disorders at baseline, after controlling for sociodemographic characteristics [40].

The face-to-face interviews were conducted by trained professional interviewers of the fieldwork agency GfK (Growth from Knowledge) Panel Services Benelux. Interviewers were selected on their experience with systematic face-to-face data collection, experience with sensitive topics and ability to achieve a good response in other studies. Fieldwork was monitored over the entire data collection period, by the NEMESIS-investigators and the fieldwork agency.

**Fig. 1:**
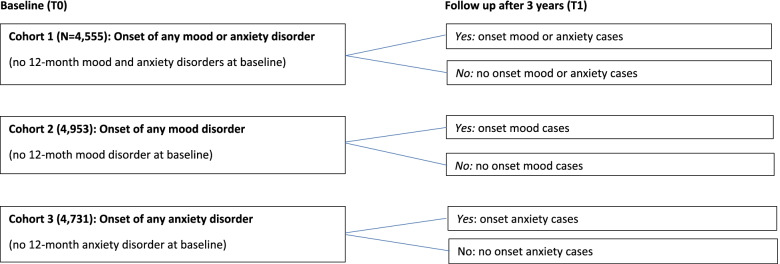
Description of the 3 study cohorts (total sample 5,303)

We studied the onset of mood (i.e. major depression, dysthymia and bipolar disorder) and anxiety disorders (i.e. Panic disorder, agoraphobia without panic disorder, social phobia, specific phobia and generalized anxiety disorder) at follow up (T1) by BMI status (underweight, normal weight, overweight, obesity) at baseline (T0). The study was carried out in three different cohorts of disorder categories (Fig. 1):

1) a cohort at risk for any mood or anxiety disorder (excluding persons with any 12-month mood or anxiety disorders at baseline; *N* = 4,555), 2) a cohort at risk for any anxiety disorder (excluding persons with 12-month anxiety disorder at baseline; *N* = 4,731) and 3) a cohort at risk for any mood disorder (excluding persons with a 12-month mood disorder at baseline; *N* = 4,953). Participants ‘at risk’ means having the possibility to become an incident case. Onset cases of a category of disorders were defined as persons who developed a disorder in a category (mood or anxiety disorder) between T0 and T1. Those who experienced a mood and/or anxiety disorders in the past twelve months at baseline were excluded. People who had experienced a mood or anxiety disorder earlier than the 12-month period before baseline were included in this study. Meaning that the onset of a disorder was either a new-onset or a recurrent disorder.

### BMI

At baseline weight and height were self-reported. BMI was calculated as weight (kg) divided by height in meters squared (m^2^). BMI was classified into four categories according to the guidelines of the WHO expert committee: underweight (BMI < 18.5), normal weight (BMI 18.5–24.9) overweight (BMI 25.0–29.9) and obesity (BMI ≥ 30).

### Mental disorders

DSM-IV diagnoses of common mental disorders were made using the Composite International Diagnostic Interview (3.0 CIDI based on DSM-IV)—a fully structured lay administered diagnostic interview. This instrument was developed and adapted for use in the World Mental Health Survey Initiative [41]. The CIDI (3.0 based on DSM-IV) used in NEMESIS-2 was an improvement on the Dutch one used in this initiative.

The disorders considered in this paper include mood disorders (i.e. major depression, dysthymia and bipolar disorder), anxiety disorders (i.e. panic disorder,agoraphobia without panic disorder, social phobia, specific phobia and generalized anxiety disorder). 'Any mood or anxiety disorder' refers to any of the above-mentioned mood or anxiety disorders.

Clinical calibration studies in various countries [42] found that the CIDI (based on DSM-IV) assesses mood, anxiety and substance use disorders with generally good validity in comparison to blinded clinical reappraisal interviews.

### Covariates

The covariates used in the analyses were fitted in three models: model 1: adjusting for gender and age; model 2: adjusting for the variables from model 1 and education (in 4 categories), ethnicity (western/non-western), employment status (paid job/no paid job), and living situation (with/without a partner); model 3 adjusting for the variables from model 1 + 2 and lifestyle factors i.e. smoking, healthy activity, psychotropic medication use, having a chronic physical disorder is the past 12 months, and baseline 12-month psychopathology: substance use disorder(i.e. alcohol/drug abuse and dependence) and if applicable, any 12- month anxiety or any 12-month mood disorder. The criteria for smoking (yes/no), were when participants smoked at least one item (e.g. cigarette, cigar, pipe) in the past month. For meeting the criteria for healthy activity standards (yes/ no) participants were asked if they were at least 5 days 30 min moderately active in an average week. This criterion is according to both national and international standards for healthy activity and the question was based on the 'International Physical Activity Questionnaire' (IPAQ; Craig et al., [43]. The use of psychotropic medication included all medication used for the treatment of mental disorders in the past 12 months (no/yes). Participants met the criteria for a chronic physical disorder (yes/no) when they had one or more chronic physical disorders, such as asthma, COPD, stomach ulcer, severe bowel disorder, hypertension, diabetes mellitus, a severe cardiac disease or myocardial infarction, and stroke (cerebral infarction or haemorrhage) or its consequences, treated or monitored by a medical doctor in the previous 12 months, assessed with a standard checklist.

### Analysis

All analyses were performed with STATA version 12.1, using weighted data to correct for differences in the response rates in several sociodemographic groups at both waves and differences in the probability of selection of respondents within households at baseline. Robust standard errors were calculated in order to obtain correct 95% confidence intervals and *p*-values [44].

First, sociodemographic -and health characteristics of the at-risk group for the onset of a mood or anxiety disorder were calculated (i.e. among those without baseline 12-month mood and anxiety disorder), in total and across BMI group, using descriptive analyses (Table 1). Second, logistic regression analyses were performed to study the longitudinal associations between BMI and the onset of mood and anxiety disorders after adjustment for several possible confounders (Table 2). In model 1, the associations were adjusted for gender and age. In model 2, these were adjusted for all sociodemographics. In model 3, the associations were additionally adjusted for lifestyle factors, psychotropic medication use, physical disorder and baseline 12-month substance abuse disorder. Next to that analysis predicting the onset of any mood disorder, were additionally controlled for baseline 12-month anxiety disorder. Analysis predicting the onset of any anxiety disorder were also controlled for baseline 12-month mood disorder.

**Table 1:** Sociodemographic characteristics and physical health at baseline of the cohort without baseline 12-month mood and anxiety disorder, in total and across BMI group at baseline (*N* = 4,555), in weighted column percentages or mean and standard errors

	Total	Underweight	Normal	Overweight	Obese	*P* value
n (%)	4,555 (100)	74 (2.0)	2387 (53.0)	1541 (32.8)	553(12.2)	
***Sociodemographic characteristics***						
Female gender	47.3	68.6	52.1	36.4	52.0	.0000 ^b,d,f^
Age at interview						.0000 ^a,b,c,d,e^
18–24	11.5	51.9	15.3	5.6	4.4	
25–34	19.8	15.3	24.0	16.0	12.7	
35–44	24.4	11.3	24.8	24.1	25.4	
45–54	23.3	14.8	19.9	27.0	29.6	
55 +	20.9	6.5	16.0	27.3	27.9	
Mean age	41.9	30.6 (1.64)	39.3 (0.45)	45.2 (0.46)	46.4 (0.77)	
Education						.0000 ^d,e,f^
Primary education	6.9	8.5	4.1	8.4	14.4	
Lower secondary	21.6	15.5	20.3	22.9	24.2	
Higher secondary	41.9	47.6	41.8	41.7	42.6	
Higher professional/university	29.6	28.3	33.8	27.0	18.7	
Non-western ethnicity	5.8	3.0	6.2	5.8	5.1	. 7556
No paid job	21.9	33.3	20.4	21.1	29.1	.0014 ^a,b,e,f^
Living without a partner	30.5	70.8	36.2	23.0	19.5	.0000 ^a,b,c,d,e^
***Physical and mental health characteristics***						
Smoking last month	29.2	37.3	30.3	28.0	25.9	.1612
Physical active	44.0	40.2	47.1	41.2	38.2	.0080 ^d,e^
Any physical disorder	32.0	26.3	26.8	34.9	48.1	.0000 ^c,d,e,f^
Psychotropic medication use	3.1	1.9	2.7	3.2	4.9	.1984
Any substance use disorder	4.4	3.8	5.7	3.4	1.6	.0016 ^d,e,f^

^a^ underweight versus normal was significantly different (*P* < .05)

^b^ underweight versus overweight was significantly different (*P* < .05)

^c^ underweight versus obese was significantly different (*P* < .05)

^d^ normal versus overweight was significantly different (*P* < .05)

^e^ normal versus obese was significantly different (*P* < .05)

^f^ overweight versus obese was significantly different (*P* < .05)

**Table 2:** Onset of mood and anxiety disorder at follow-up in total and across specific BMI group at baseline (*N* = 4,555), in weighted percentages and adjusted odds ratios

n (%)	N at risk	N onset	%	Adj OR ^a^	Adj OR ^b^	Adj OR ^c^
* 3-year onset of mental disorders*						
Onset of any mood or anxiety disorder	4555	385	9.2			
**BMI**						
Underweight	74	5	6.0	.42 [.15,1.16]	.39 [.14,1.11]	.43 [.15,1.27]
Normal	2387	214	9.6	Ref	Ref	Ref
Overweight	1541	107	7.3	.98 [.69,1.38]	.93 [.67,1.28]	.95 [.68,1.32]
Obese	553	59	12.7	**1.68 [1.07,2.65]**	**1.57 [1.02,2.42]**	**1.71 [1.11,2.62]**
* P for trend*				***.003***	***.005***	***.003***
Onset of any mood disorder	4953	305	6.8			
**BMI**						
Underweight	79	5	7.9	.91 [.30, 2.73]	.84 [.27, 2.57]	.80 [.30, 2.19]
Normal	2609	164	6.5	Ref	Ref	Ref
Overweight	1664	88	6.5	1.24 [.85, 1.80]	1.17 [.84, 1.64]	1.12 [.82, 1.53]
Obese	601	48	8.5	**1.56 [1.05,2.33]**	1.43 [.97,2.11]	**1.46 [1.02,2.09]**
* P for trend*				***.005***	***.017***	***.020***
Any anxiety disorder	400	65	18.2			**2.92 [1.93, 4.42]**
Any substance use disorder	188	24	12.3			1.69 [.84, 3.41]
Onset of any anxiety disorder	4731	252	6.0			
**BMI**						
Underweight	79	4	5.1	.52 [.16,1.73]	.47 [.14,1.63]	.44 [.11,1.83]
Normal	2483	142	6.5	Ref	Ref	Ref
Overweight	1595	66	4.1	.84 [.55,1.28]	.80 [.52,1.22]	.89 [.56,1.43]
Obese	574	40	9.0	**1.81 [1.04,3.15]**	1.71 [.99,2.93]	**1.86 [1.03,3.37]**
* P for trend*				***.013***	***.019***	***.008***
Any mood disorder	175	38	19.9			**2.42 [1.47, 3.98]**
Any substance use disorder	172	19	13.1			1.58 [.73, 3.40]

Onset refers to first-incidence or recurrence of a mood or anxiety disorder

^a^ adjusted for gender, age

^b^ adjusted for model 1 and for education, ethnicity, job status, living situation

^c^ adjusted for model 2 and for smoking status, physical activity, psychotropic medication use, physical disorder, baseline 12-month other psychopathology

*Ref* Reference category

Bold: Significant OR at the 0.05 level, 2-sided test

## Results

### Description of the sample

A total of three cohorts were investigated in this study. The first cohort predicts the onset of any mood or anxiety disorder (*N* = 4,555), therefore persons with a 12-month mood or anxiety disorder at baseline were excluded. The second cohort predicts the onset of any mood disorder (*N* = 4,953), hence persons with a 12-month mood disorder at baseline were excluded. The prevalence rates of anxiety disorders and substance abuse in this sample were 18.2% and 12.3% respectively. The third cohort predicts the onset of any anxiety disorder (*N* = 4,731), thus persons having a 12-moth anxiety disorder at baseline were excluded. The prevalence rates of mood disorders and substance abuse in this sample were 19.9% and 13.1% respectively.

The characteristics the three cohorts were very comparable, therefore we only present the sociodemographic- and health characteristics of the study cohort predicting the onset of any mood or anxiety disorder. Of this cohort, 47.3% was female and the mean age was 41.9 years. A total of 553 (12.2%) respondents were obese, 1,541 (32.8%) were overweight, 74 (2.0%) were underweight and 2,387 (53.0%) had a normal weight. Respondents with obesity were more often older, lower educated, had no paid job, were living with a partner, were physical inactive, had a physical disorder and a substance use disorder compared to respondents with normal weight. Respondents with overweight were more often male, older, lower educated, living with a partner, physical inactive and had a physical disorder compared to respondents with normal weight. Compared to respondents with normal weight, respondents with underweight were more often younger, had no paid job and were living without a partner (see Table 1).

There was no significant difference between BMI level and ethnicity, smoking and psychotropic medication use. Attrition rate was not significantly associated with all main categories and specific 12-month mental disorders at baseline, after controlling for sociodemographic characteristics [39].

### The association between BMI and 3-year onset of any mood or anxiety disorders

We investigated the relationship between BMI categories (underweight, overweight and obesity) and the incidence of any mood or anxiety disorder (N at risk = 4,555) (Table 2). Results showed that respondents with obesity compared to those with normal weight, have an increased risk of the onset of any mood or anxiety disorder adjusting for age and gender (model 1: OR = 1.68; 95% CI: 1.07–2.65). This relationship remained significant after additionally adjusting for other sociodemographic factors (model 2; OR = 1.57; 95% CI: 1.02–2.42), lifestyle factors, presence of a chronic physical disorder, psychotropic medication use and presence of other psychopathology (i.e. substance use disorder) 12 months prior to baseline (model 3; OR = 1.71; 95% CI: 1.11–2.62). The OR of the underweight category was low and non-significant. We must note that only 2% (*N* = 74) of the respondents were underweight; so due to lack of power it is not possible to draw any conclusions about this category. Moreover, no significant relationship was found between the BMI category overweight compared to those with normal weight and the onset of any mood or anxiety disorder.

A significant P for trend was found in model 1, indicating that there was a dose–response effect of the continuous BMI scores on any mood or anxiety disorder, which remained significant after adjusting for variables in model 2 and 3 (OR = 1.06; 95% CI: 1.02 = 1.10; *p* < 0.01).

### The association between BMI and the 3-year onset of mood disorders, and the association between BMI and the 3-year onset of anxiety disorders

We investigated the association between BMI and onset of the specific categories of disorders (i.e. any mood disorder (N at risk = 4,953) and any anxiety disorder (N at risk = 4,731). The results showed that respondents with obesity have an increased risk of the onset of any mood disorder compared to respondents with normal weight, adjusting for gender and age (model 1: OR = 1.56; 95% CI: 1.05–2.33). This association remained significant in the fully adjusted model (model 3: OR = 1.46; 95% CI: 1.02–2.09), but not in model 2 (*p* = 0.074). However, the OR for obesity in model 2 did not differ much from those in model 1 and 3.

Respondents with obesity at baseline also had an increased risk of the onset of any anxiety disorder compared to those with normal weight when adjusted for age and gender (model 1: OR = 1.81; 95% CI: 1.04–3.15). Again, this association remained significant in the fully adjusted model (model 3: OR = 1.86; 95% CI: 1.03–3.37), but not in model 2 (*p* = 0.053). Also, now the OR of model 2 did not differ that much from model 1 and 3. No significant relationship was found between the other BMI categories (underweight and overweight) and the onset of any mood disorder as well as any anxiety disorder.

We found a significant P for trend of continuous BMI on any mood disorder as well as any anxiety disorder. These results indicate that there was a dose–response effect of the continuous BMI level on both any mood disorder and any anxiety disorder, which remained significant after adjustment in all models 1, 2 and 3 (*p* < 0.05). Additionally, we have looked at sex-specific differences in all three cohorts. However, we found no significant results.

## Discussion

### Key findings

This prospective study among the adult general population (18–64 years) found evidence that obesity predicts the onset (i.e. first-onset or recurrence) of any mood or anxiety disorder after adjustment for potential confounding socioeconomic and lifestyle variables, psychopathology at baseline, psychotropic medication use and physical disorders. We tested this association in three different cohorts (i.e. at risk groups): any mood or anxiety disorders, any mood disorder, and any anxiety disorder), but the results did not differ substantially. Next to that we found a dose response relationship of BMI on the onset of both mood and anxiety disorders. This study did not find evidence for an association between underweight as well as overweight at baseline and the onset of a mood and/or anxiety disorder in the general population. Hence, we did not find evidence for a U-shaped association between BMI and mood and anxiety disorders.

### Discussion of findings and implications

Up till now there have not been many high-quality cohort studies investigating the risk of the incidence of DSM-IV mood and anxiety disorders with BMI status at baseline [13, 14, 17]. Especially the studies on the incidence of anxiety disorders are scarce [13]. The findings of the current study of an increased risk of the onset of a mood disorder in persons with obesity compared to those with normal weight was consistent with previous meta-analysis on the relation between BMI status and mood disorders in the adult general population [13, 14]. Finding a positive effect of obesity at baseline and the onset of anxiety disorder was also in line with previous studies [32, 33]. However, the study of *Bjerkeset* et al. (2008) found only an increased risk of anxiety symptoms in men, and not in women. *Kasen* et al. (2008) studied specific the onset of generalized anxiety disorders (GAD) and major depressive disorder (MDD). He found an elevated risk for both GAD and MDD in persons with obesity at baseline found an elevated risk. More research is needed investigating the onset of specific DSM diagnoses (e.g. GAD and MDD) in specific subgroups (e.g. gender, age) in persons with obesity at baseline. Due to power problems, we were not able to investigate specific mood and anxiety disorders in this study and had to categorize them. Next to that, we were unable to investigate specific subtypes of depression in the current data set (NEMESIS-2). Previous studies have shown that mainly atypical depression is associated with obesity [45–47] Future high-quality longitudinal research which focusses on specific diagnosis and specific subsamples samples is needed to further disentangle the relation between BMI and onset risk of mood and anxiety disorders.

In this study no relationship was found between overweight and both mood and anxiety disorders. These results are not in line with a previous meta-analysis that found an elevated risk for major depressive disorder in persons with overweight compared to persons with normal weight [13]. A more recent meta-analysis by *Jung* et al. (2017) that contained more high-quality studies compared to the previous meta-analysis by *Luppino* et al. (2010) found that the relationship between overweight and onset of mental disorders was only found in women, not in men. We however, did not find sex-specific differences, possible due to power problems in our study.

Based on the results of a recent meta-analysis by *Jung* et al. (2017) we expected to find a U-shaped association between BMI levels and the onset of mood and anxiety disorders. However, we did not find an indication for an association between underweight and the onset of mood and anxiety disorders. The fact that we did not find a significant result might be due to the small percentage (2%) of people with underweight in the sample and therefore there was a lack of power to investigate this properly.

The effect of obesity at baseline on the onset mood and anxiety disorders found in this study might be explained by several psychological and biological factors mediating or moderating the association. The role of psychological factors might be the influence of stigma and weight discrimination. Being overweight or obese is still stigmatized, which is in turn associated with body image dissatisfaction, low self-esteem, anxiety and depression [18, 48, 49]. We know from previous studies, that biological markers also play an important role. Common genes in depression and obesity could be a potential mechanism explaining the association. For instance, depression had shown to increase the effect of the FTO gene on BMI [23]. Next to that, inflammatory markers—often seen in persons with obesity—are also associated with depression [16, 19, 21, 22]. In addition, chronic stress leading to dysregulation of the hypothalamic–pituitary–adrenal-axis (HPA –axis) is leading to elevated levels of cortisol, which is in turn associated with both obesity, anxiety and depression [16, 19, 20]. So, when people have obesity at baseline, the onset of mood and anxiety disorders might be influenced by these negative psychological factors on one hand and disturbed biological processes on the other hand. However, we were unable to investigate these factors in the current study. Future research should take these mediating and moderating factors into account to further disentangle the underlying mechanisms in the association between obesity and mental disorders.

### Strengths and limitations

The strengths of this study were that our sample consists of a relatively large cohort of adults in the general population, that was examined twice over a period of 3 years. Furthermore, mood and anxiety disorders were assessed with a well validated standardized psychiatric assessment interview [41]. Furthermore, we assessed a broad array of common mental disorders and were able to control for comorbid psychopathology at baseline. Moreover, we were able to control for a broad range of potential critical confounding variables including sociodemographics, lifestyle factors, psychotropic medication use and physical disorders. Next to that, compared to previous studies we able to better investigate the onset of mental disorders in persons with unfavorable BMI categories. There were limitations to this study that must be mentioned as well. First, the data of NEMESIS-2 are representative for most parameters of the Dutch population. However, people with an insufficient mastery of the Dutch language, those with no permanent residential address and the institutionalized, are underrepresented in this sample. Hence, our findings cannot be generalized to these groups. Second, even though the total study sample of NEMESIS-2 is relatively large, we were not able to investigate first-onset and recurrence of mood and anxiety disorders separately, nor the incidence of specific mood and anxiety diagnosis, because of power problems. Moreover, we were not able to perform analysis on specific subgroups such as atypical depression which has shown to be associated with obesity in previous studies [45, 46]. Third, BMI was self-reported which might have led to reporting bias of weight status, showing under-reporting for weight and over-reporting for height [50]. In this case, the actual associations will be even stronger. On the other hand, according to validation studies it is unlikely that bias in self-reported BMI is affecting conclusions about associations in prospective epidemiological studies [51, 52].

Fourth, the number of underweight people in this study cohort is small (*n* = 74; 2.0%) which probably resulted in non-significant associations with later onset of mood and/or anxiety disorders. This implies we cannot draw reliable conclusions about these associations. Lastly, in our sample we excluded only participants with a 12-month mood and anxiety disorder, not with a lifetime disorder. Therefore, the onset cases are both new and recurrent cases and we are not able to distinguish between these groups in this study.

In conclusion, this population-based study gives evidence for the risk the onset of any clinical diagnosis of mood and anxiety disorders in persons with obesity compared to persons with normal weight. These findings suggest that guidelines for obesity treatment need to address the management of comorbid mood and anxiety disorders. In dietary practice, patients with obesity should be informed about their increased risk of developing a mental disorder. In clinical practice, care providers should monitor mental health in persons with obesity in order to prevent and treat mental health problems in this group.

These findings may have a major public health importance because both obesity and mood and anxiety disorders are linked to morbidity and mortality. Future large cohort high quality longitudinal studies especially focusing on subgroups, specific types of mood and anxiety disorders and underlying biological and psychological factors are recommended to further disentangle the relation between BMI categories and mental disorders.

## Data Availability

The data on which this manuscript is based are not publicly available. However, data from NEMESIS-2 are available upon request. The Dutch ministry of health financed the data and the agreement is that these data can be used freely under certain restrictions and always under supervision of the Principal Investigator (PI) of the study. Thus, some access restrictions do apply to the data. The PI of the study is second author of this paper and can at all times be contacted to request data. At any time, researchers can contact the PI of NEMESIS-2 and submit a research plan, describing its background, research questions, variables to be used in the analyses, and an outline of the analyses. If a request for data sharing is approved, a written agreement will be signed stating that the data will only be used for addressing the agreed research questions described and not for other purposes.
